# The Acoustic Environments in Which Older Adults Wear Their Hearing Aids: Insights From Datalogging Sound Environment Classification

**DOI:** 10.1044/2018_AJA-18-0061

**Published:** 2018-12-06

**Authors:** Larry E. Humes, Sara E. Rogers, Anna K. Main, Dana L. Kinney

**Affiliations:** a Department of Speech and Hearing Sciences, Indiana University, Bloomington

## Abstract

**Purpose:**

This report presents data on the acoustic environments in which older adults with age-related hearing loss wear their hearing aids.

**Method:**

This is an observational study providing descriptive data from 2 primary datasets: (a) 128 older adults wearing hearing aids for an average of 6 weeks and (b) 65 older adults wearing hearing aids for an average of 13 months. Acoustic environments were automatically and continuously classified about every 4 s, using the hearing aids' signal processing, into 1 of 7 acoustic environment categories.

**Results:**

For both groups, older adults wore their hearing aids about 60% of the time in quiet or speech-only conditions. The automatic classification of sound environments was shown to be reliable over relatively short (6-week) and long (13-month) durations. Moreover, the results were shown to have some validity in that the obtained acoustic environment profiles matched a self-reported measure of social activity administered prior to hearing aid usage. For a subset of 56 older adults with data from both the 6-week and 13-month wear times, the daily amount of hearing aid usage diminished but the profile of sound environments frequented by the wearers remained stable.

**Conclusions:**

Examination of the results from the automatic classification of sound environments by the hearing aids of older adults provides reliable and valid environment classifications. The present data indicate that most such wearers choose generally favorable acoustic environments for hearing aid use.

According to the U.S. Census Bureau, 43.1 million Americans were aged 65 years and over in 2012, and by 2050, this population is expected to nearly double in size to 83.7 million (Ortman, Velkoff, & Hogan, 2014). This report further states that, in terms of percentage of the U.S. population, adults aged 65 years and over represented 13.7% of Americans in 2012 and will represent 20.9% by 2050, achieving 20% representation as soon as 2030. Clearly, the health issues confronting this burgeoning segment of the American population are an immediate concern and will continue to be a concern for the foreseeable future.

Hearing loss is the third most prevalent chronic health problem experienced by Americans aged 65 years and over (Hearing Loss Association of America, 2017). Various estimates place the prevalence of hearing loss among older Americans at 30%–40%, depending, in large part, on how hearing loss has been defined (Campbell, Crews, Moriarty, Zack, & Blackman, 1999; Cruickshanks, Zhan, & Zhong, 2010). One of the most common communication complaints among those aged 65 years and over with age-related hearing loss (ARHL) is that they can hear speech but cannot understand it. Given the typical mild–moderate sensorineural hearing loss experienced by most older adults (International Organization for Standardization, 2000), hearing aids represent the most common and most appropriate intervention.

The everyday benefits provided by hearing aids to those with ARHL will depend critically on the acoustical environments in which the older adult is immersed daily. Both self-report outcomes and objective measures of speech recognition demonstrate greater hearing aid benefit in quieter environments (e.g., Humes, Ahlstrom, Bratt, & Peek, 2009; Humes, Garner, Wilson, & Barlow, 2001; Kochkin, 2010; Larson et al., 2000). Thus, when projecting the hearing aid benefits to be experienced by the typical older adult with ARHL, it is important to have a realistic estimate of the common acoustical environment, or range of environments, experienced.

There has been a long-standing interest in better establishing the typical acoustical environments experienced by older adults, including those with ARHL. Walden, Surr, Cord, and Dyrlund (2004), for example, desiring to better understand hearing aid user's preferences for directional microphone activation, had 17 participants log acoustic events three times per day over a 4-week period. Participants used pencil and paper to record binary choices about several acoustical features of the environment, such as noise present or absent, signal near or far, signal at front or other location, and so forth. The 17 participants were older adults with a mean age of 70.8 years, had bilateral moderate sloping hearing loss as a group, and were experienced hearing aid wearers. Importantly, only recorded responses for “active listening” situations, those for which they were engaged in listening to speech, music, or sounds of nature, were included in the analyses reported. Of the 1,599 active listening entries, 37% were noted as “noise absent” periods with the balance being “noise present” periods. Of the noise-present periods, however, the majority, representing 36% of the 1,599 total entries, were conditions likely to have favorable speech-to-noise ratios (near signal at front with noise present but from other nonfrontal directions). Thus, about three fourths of the everyday listening conditions involved favorable acoustical conditions; conditions for which hearing aids would likely be of help. Moreover, when ranked by estimated time spent in that type of listening condition, five of the top eight were noise-absent conditions, and participants spent about 6.6 hr per day in such active listening conditions, excluding time spent listening to the radio or watching television.

Using an approach somewhat similar to Walden et al. (2004), Keidser (2009) reported data from 28 older adults (*M* = 74 years) with moderate hearing loss who were experienced hearing aid users. For the 606 diary entries obtained, 26% were for “speech in quiet,” and an additional 15% were for “mostly quiet,” whereas 24% were for “speech in noise.” Banerjee (2011) implemented a digital version of the pencil-and-paper environment classification in a study of nine older adults, eight of whom were experienced hearing aid wearers. Although the focus of this study was the everyday use of multiple programs and the volume control, data reported on the acoustical environments encountered over a 4- to 5-week period agree with those of Walden et al. (2004) and Keidser (2009).

Rather than have the participants record key acoustical features of daily experiences, Wagener, Hansen, and Ludvigsen (2008) recorded brief samples of the acoustical environment for subsequent laboratory analysis. They had 20 experienced hearing aid wearers, with a wide range of ages, backgrounds, and hearing loss, “record different situations from your daily life for 5-10 minutes each” (p. 353) using head-worn microphones and digital audiotape recorders. Each individual sampled 5-min situations for a 3- to 4-day period, varying in total recording length from 46 to 121 min per person, with a total of 349 individual listening situations selected by the investigators for detailed analysis. Slightly more than half (50.7%) of the analyzed samples involved conversation, with 11.5% occurring “without background noise,” 17.8% “with background noise-2 persons,” and 10.3% “with background noise > 2 persons” (p. 354). Smeds, Wolters, and Rung (2015) subsequently analyzed these same recorded materials to estimate typical signal-to-noise ratios experienced by the hearing aid wearers. Signal-to-noise ratios are known to vary dramatically with noise level, being larger at low (< 50 dBA) noise levels and near 0 or negative at high noise levels (> 75 dBA; e.g., Pearsons, Bennett, & Fidell, 1977), and this was confirmed by Smeds et al. (2015). For moderate noise levels of 55–60 dBA, Smeds et al. (2015) observed signal-to-noise ratios of 8–12 dB for the conditions recorded originally by Wagener et al. (2008). These recorded samples also suggest that adults with impaired hearing wear hearing aids in generally favorable acoustical conditions, conditions for which hearing aids are beneficial (e.g., Humes et al., 2009; Humes et al., 2001; Larson et al., 2000).

One of the limitations of the acoustical data from Wagener et al. (2008) and Smeds et al. (2015) is that the recordings represent very brief glimpses of the hearing aid wearer's typical daily auditory experiences. Wu and Bentler (2012) addressed this by having their participants carry a noise dosimeter for 14 hr/day over a 7-day period, yielding a sample of 98 hr for the week of recording. They were instructed, such as the participants in Walden et al. (2004), to make closed-set choices about the listening activity and environment for those listening situations lasting at least 10 min. Participants (*N* = 27) all had mild–moderate bilaterally symmetrical sensorineural hearing loss and a mean age of 66 years, with 20 of the 27 being hearing aid users. Across the 1,267 entries covering 2,032 hr of dosimeter recordings, 61.2% involved speech, including conversations and listening to media, such as television. The three most common situation/environment combinations noted by the participants were little or no speech listening at home (24%), speech listening via media at home (21%), and small group conversation at home (10%). A subgroup of adults older than 65 years (*n* = 14) tended to experience quieter listening situations and was less active socially than a subgroup younger than 65 years (*n* = 13) with comparable hearing loss. The differences in acoustic environments between these two subgroups of older adults, however, tended to be small.

Although the study by Wu and Bentler (2012) greatly extended the duration of the recorded samples, the participant still decided when the listening situation and environment for a specific acoustic event should be logged. To overcome this selection bias, investigators have coupled acoustical recordings with efficient classification of several features of the sound environment at randomly selected moments in time (Timmer, Hickson, & Launer, 2017a, 2017b; Wu et al., 2018). These studies have made use of ecological momentary analysis (EMA) to sample intervals while the wearers go through their everyday activities, where the intervals are either randomly selected or self-selected. For each sample, acoustic recordings are obtained roughly coincident with the wearer's completion of various items in the EMA. The nature of the items used in the EMA can vary as desired but typically include some categorization of the listening activity, acoustic environment, and benefit or difficulty experienced. Because the samples may be chosen randomly and frequently (e.g., every 2 hr, on average), there is less potential for selection bias by the participant during the recording. One of the striking features of the findings in these recent studies, somewhat consistent with the earlier studies summarized above, is the large proportion of relatively easy listening conditions recorded. In Wu et al. (2018), for example, 43% of the recordings were classified as being in relative “quiet” with speech present in low levels of noise. Note, however, that here, as in several of the prior studies reviewed above, those samples or situations for which the wearer of the device was not “actively engaged” (p. 297) in conversation or listening, based on the researchers' post hoc impression of the recordings, were eliminated from further analyses. Improvement in performance for active listening situations is clearly the goal for hearing aids, but excluding all other recordings presents a biased sample of the hearing aid user's daily acoustical experiences. Another limitation of the preceding studies is that all have been based on sampling small portions of the person's everyday listening experience, whether this is wearer directed or randomly sampled. Finally, except for Timmer, Hickson, and Launer (2017b), at least 75% of the participants in each of the foregoing studies of everyday sound environments were experienced hearing aid wearers. Much less information is available for the typical listening environments of new hearing aid users.

Many contemporary hearing aids include sophisticated sound environment classification systems making use of the digital signal processing resources available. It has long been thought that such sound environment classification systems could be used to automatically guide the application of various signal-processing algorithms in the hearing aid, including noise reduction and directional systems (e.g., Büchler, Allegro, Launer, & Dillier, 2005; Kates, 1995). An advantage of the use of such systems to gain insights into the acoustical environments experienced typically by the wearer is that they obtain frequent samples during everyday use of the hearing aids and provide a cumulative tally of the acoustical features of the environments detected.

Groth and Cui (2017) provided a description and validation of one hearing aid–based sound classifier implemented in a commercial product line and compared the accuracy of that classifier with several others available in contemporary hearing aids. In this observational study, we provide data obtained from the same sound classifier described by Groth and Cui (2017) over both short (~6 weeks) and long (~1 year) time periods. To date, most such descriptive data have been obtained for durations up to 6 weeks. Does the nature of the acoustical environment encountered by new hearing aid wearers change over time, from 6 weeks to 1 year? Specifically, this report provides descriptive data from (a) 128 hearing aid wearers using their hearing aids for approximately 6 weeks representing classification of nearly 71,000 hr of acoustic input and (b) 65 hearing aid wearers wearing their aids for about 1–2 years (*Mdn* = 13 months) representing more than 240,000 hr of acoustic input classified. Not only are these data much more extensive than those published in prior studies but they are also exclusively from new hearing aid wearers. In this approach, complementary to those pursued previously as reviewed above, we obtain continuous analysis of the acoustic environment experienced by older adults with ARHL wearing hearing aids over extended periods of time, but the device is automatically classifying the acoustic environment rather than the participant doing so periodically with pencil and paper or via smartphone (as in EMA).

## Method

**Figure 1.**
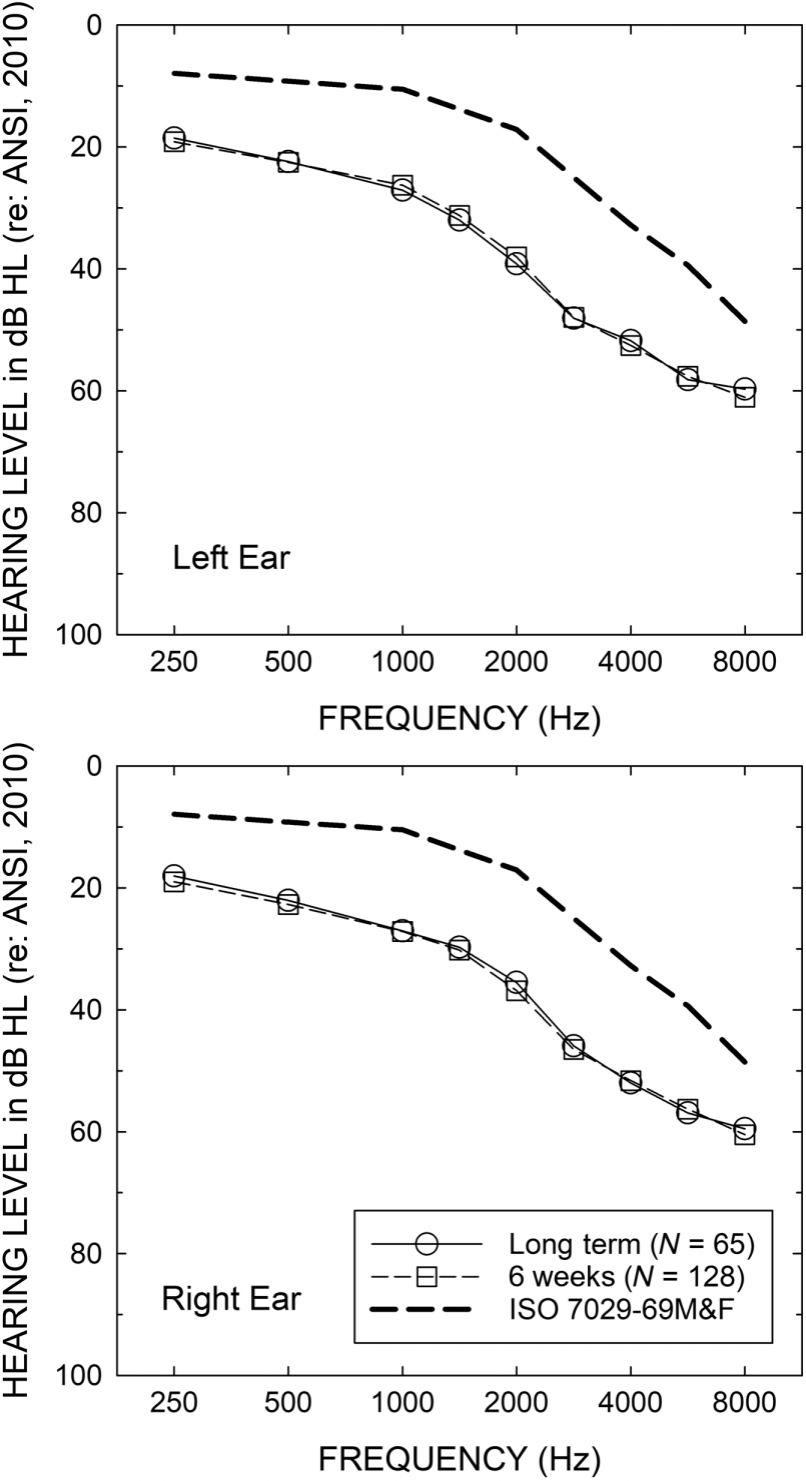
Median air conduction pure-tone thresholds for the left (top) and right (bottom) ears for the group of 128 older adults wearing hearing aids for an average of 6 weeks (squares) and the group of 65 older adults wearing hearing aids for an average of 13 months (“long-term,” circles).

There were two study samples from which the environmental sound classification data were obtained. The larger group provided such data over a shorter period, on average for about 6 weeks of hearing aid usage, and was composed of 128 adults, 67 males and 61 females, having a mean age of 69.4 years (ranging from 54 to 80 years). The median education level was a master's degree, and all 128 participants had at least completed high school. About two thirds (68%) of the participants were retired at the time of study enrollment. All participants were from southcentral Indiana. The median audiograms for the left and right ears are provided in Figure 1. All 128 participants were first-time hearing aid wearers. Additional demographic information for this larger group appears in Table 1.

**Table 1.** Demographics for two datasets examined in this report.

Measure	6-week group (*N* = 128)		Long-term group (*N* = 65)	
	*M*	*SD*	*M*	*SD*
Age (years)	69.4	5.6	69.1	5.5
Loss duration (years)	8.1	9.0	6.6	5.8
Time delayed (years)	4.3	5.7	3.4	3.3
Duration retirement (years)	6.4	7.5	6.1	7.5
SRT right (dB HL)	23.9	9.8	23.1	8.6
SRT left (dB HL)	23.8	8.8	23.6	9.1
WRS right (%)	90.1	9.5	91.0	7.7
WRS left (%)	88.8	8.5	88.0	9.5
MMSE	28.6	1.4	28.7	1.2
HHIE	30.4	14.9	29.5	14.6
CST (rau) SF 65/+3	57.8	22.9	54.8	22.7

*Note.* SRT = speech reception threshold; WRS = word-recognition score; MMSE = Mini-Mental State Examination; HHIE = Hearing Handicap Inventory for the Elderly; CST = Connected Speech Test; rau = rationalized arcsine units; SF = sound field.

The second study sample was composed of 65 older adults, 56 of whom were also among the 128 participants described above. These 65 participants had agreed to return for a follow-up visit from 1 to 3 years (*Mdn* = 13 months) after the original hearing aid fitting. This subgroup was composed of 37 males and 28 females. The educational level and retirement status of this subgroup were identical to those of the larger group described above. Additional demographic information for this subgroup also appears in Table 1.

All participants included in these analyses were first-time hearing aid wearers who had either participated in a previously published randomized clinical trial (Humes et al., 2017) or a smaller scale follow-up clinical trial that has yet to be published but followed the same hearing aid selection procedures as the consumer-decides group in Humes et al. (2017). All participants were fitted with the same hearing aids bilaterally, fitted either by an audiologist or by the participant following the consumer-decides group procedures described in Humes et al. (2017). The hearing aids used in the study were all the same: GN ReSound Alera 9 mini–behind-the-ear open-fit devices. The directionality of all devices was either set to omnidirectional (for those with placebo fits in the original study) or “directional fixed” in the programming software, with the latter choice establishing a hypercardioid directional pattern for the microphones. However, for those with fixed directivity, the directional mix parameter in the software was set to “very low,” which made the hearing aids function closer to an omnidirectional microphone device over most of the frequency range. Specifically, there was no directional benefit except for the frequency range from 1500 to 3000 Hz and again at about 5500 Hz for which sounds from 180° azimuth were attenuated for an average of 5 dB relative to 0° azimuth. All hearing aids were tested in the Verifit test box (Model VF-1; Software Version 3.10.9) to ensure that they met American National Standards Institute (2009) specifications prior to programming. The function of the directional microphone systems was also verified acoustically at that time. The directionality of the microphones described above was programmed into the hearing aids by the audiologist and was not adjustable by the wearer.

The acoustic environment classification system makes use of three signal-processing detectors, one each for speech, noise, and overall level. The speech detector generates a probability that the microphone input was speech or not speech, the noise detector generates a probability that the environment was noise or not noise, and the level detector creates a probability that the broadband power level was at least 75 dB SPL or not. With three independent detectors and two choices for each, a total of eight environmental categories could be produced. However, for the not-speech and not-noise combination, indicating a quiet environment, the classification of sound level (≥ 75 dB SPL or not) is not logical, and this combination is omitted. In this report, we have designated levels < 75 dB SPL as “moderate” and those ≥ 75 dB SPL as “high.” In the end, each of the seven acoustic environment classifications is assigned a probability, and the set of seven probabilities adds to 1. As an example, a high probability of speech from the speech detector and a low probability of noise from the noise detector, together with a low probability of the level exceeding 75 dB SPL from the level detector, would indicate that this environment likely was composed of “moderate level speech only” with the other six possible environments all having relatively low probabilities. In this way, the environment classification with the highest probability is logged automatically into the hearing aid's datalogger with updates to the classification about every 4 s. In the datalogger, the values logged are in cumulative time (hours) for each of the seven acoustic environments. For example, given updates every 4 s, 15 classifications of “moderate level speech only” would amount to 1 min in that environment, and 900 such classifications would register as 1 hr in that environment. These logs are generated separately for each of the four programs to which the devices could be set (all four were programmed to serve solely as a volume control; Humes et al., 2017), and the data reported here are summed across all four programs. By dividing the cumulative time for each of the seven classified environments by the total hours of usage logged in the datalogger, we established the proportion of time the hearing aids were worn in each of those sound environments. Importantly, this automatic environment classification processing is applied to the output of the hearing aid microphone prior to any amplification or processing of that electrical signal. As a result, the classification of the acoustic environment is independent from the hearing aid–related processing of the devices. As noted, Groth and Cui (2017) have recently validated the ability of this system to accurately classify a variety of acoustic environments.

## Results and Discussion

### Data From 128 Wearers Using Devices for an Average of 6 Weeks

**Figure 2.**
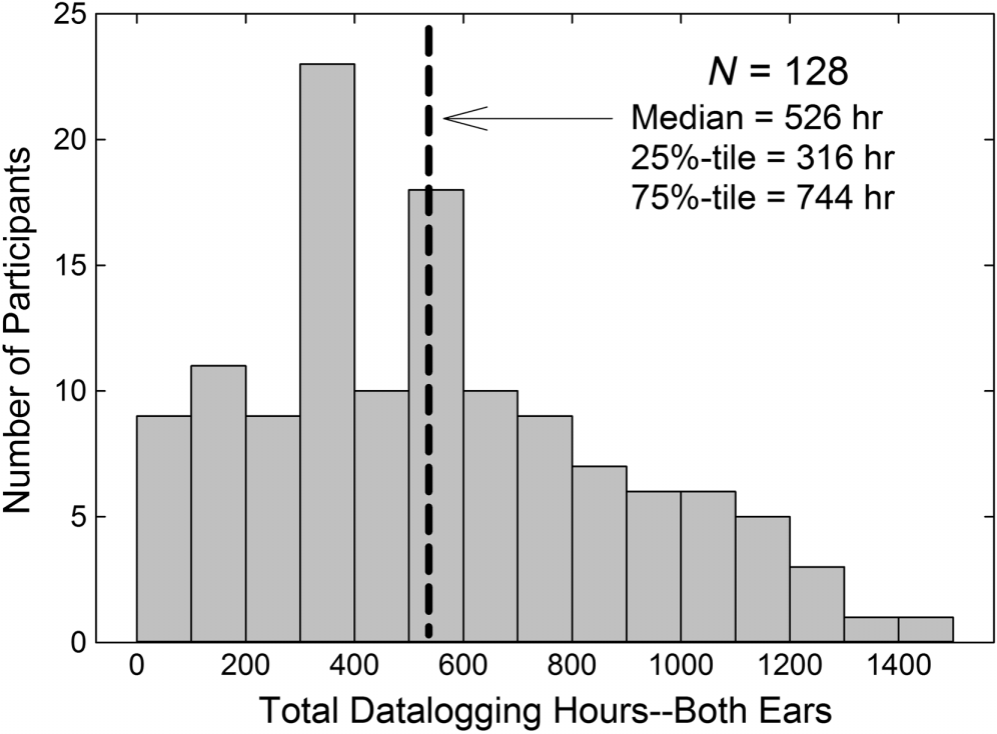
Histogram showing number of participants among the group of 128 who wore their hearing aids for a bilateral total of 0–99, 100–199, 200–299,…1,400–1,499 hr. Median value of 526 hr is shown as the heavy dashed line.

The distribution of total hours of hearing aid usage, summed across right and left ears, is shown in Figure 2. As depicted, there was a wide range of usage with a median for the 128 wearers (256 hearing aids) of 526 hr. Given a roughly even split between right (*Mdn* = 250 hr) and left (*Mdn* = 265 hr) ears and average daily usage of about 6.5 hr/day (Humes et al., 2017), the median usage of 526 hr in Figure 2 translates to an average of about 40.5 days (526 hr per pair, or 263 hr per aid, divided by 6.5 hr per aid per day) or 6 weeks, which was the duration of the clinical trial period. The hours of usage during this 6-week trial, however, clearly varied from wearer to wearer as illustrated in Figure 2. There was no significant difference between the mean hours of usage for the right and left devices, paired *t*(127) = −0.47, *p* = .64, and the correlation was strong (*r* = .85, *p* < .001).

**Figure 3.**
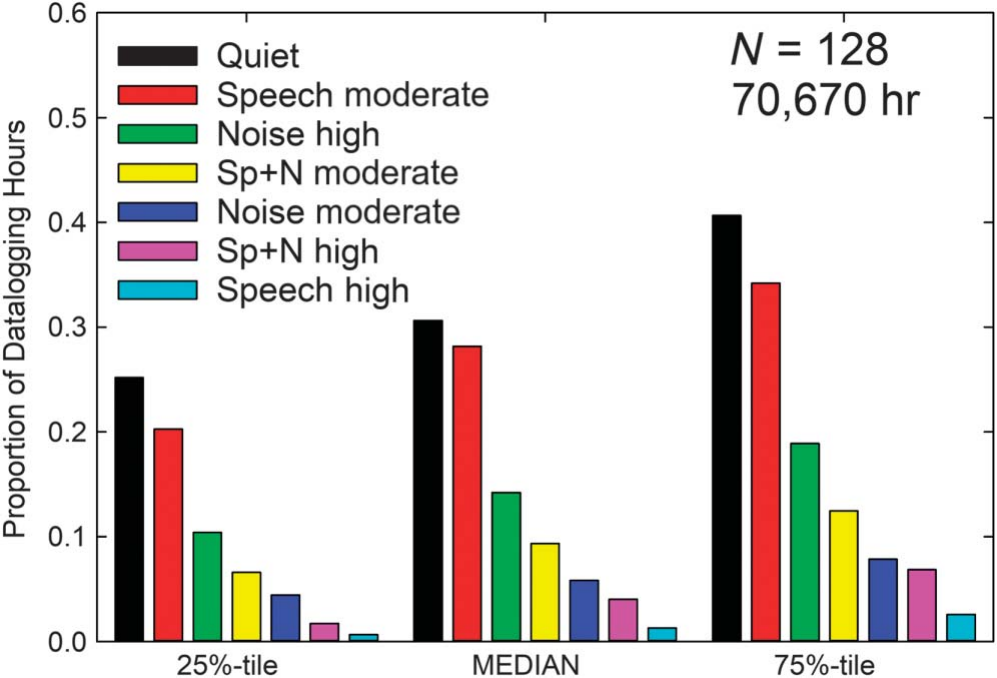
Medians and quartiles for the proportion of total hours the hearing aids were worn in each of seven acoustic environments as classified by the devices automatically for the 128 wearers who logged a total of 70,670 hr (summed across ears and participants). Sp+N = speech and noise.

Figure 3 provides the median and quartiles for the proportion of time the devices classified the acoustical environment into one of the seven possible categories. As shown, the cumulative data from the 128 wearers wearing two devices for an average of about 6.5 hr/day for about 6 weeks amount to a total of 70,670 hr logged. The median data indicate that the typical wearer spent about 60% of the time in environments that were quiet or included moderate (< 75 dB SPL) speech only with high levels (≥ 75 dB SPL) of speech in quiet or in noise being the least frequent acoustic environments, generally occurring less than 5% of the time.

The reliability of the datalogging can be assessed by correlating the results for the devices worn on the right and left ears. The devices are independent, but the environments in which they are worn are not, at least in most typical situations. That is, in most cases, the acoustic environment of the left device should be very similar, if not identical, to that of the right device. Thus, if the hours and the classifications are reliable, we would expect strong correlations between the results of the right and left ears. As noted above, this was the case for the total hours of usage for right and left devices (*r* = .85). For the environmental classifications, data from the clinical trial of Humes et al. (2017) for right and left ears separately were not consistently archived. Rather, given that such datalogging details were not the focus of that study, the bilateral sum of the environmental sound classifications was routinely archived and forms the primary basis of the datasets used here. As a result, separate results for right and left devices were only available for 60 of the 128 wearers or 120 devices. A subsample of 47% (60/128), however, is appropriate for reliability analyses. For 52 of the 60 (87%) right–left pairs, the Pearson correlations for the usage pattern proportions across the seven environmental classifications were ≥ .90. Further, of the eight pairs not achieving this high criterion correlation, five had interear correlations > .70. The remaining low correlations (*r* < .70) between right and left device environmental classification proportions were all for those who had low usage during the 6-week trial. Based on these correlations between the classification proportions made by the right and left devices in 60 device pairs, about half of the sample, the results for the sample of 128 wearers are considered reliable.

Given that the data are reliable and we have such classification data from 128 wearers, we wondered whether there were individual differences in the patterns of acoustical environments in which hearing aid wearers wore their devices, and if so, what variables or factors might predict those different usage patterns. To examine differences in patterns among the 128 wearers, we made use of a two-step cluster analysis with a log-likelihood distance clustering metric, a Schwarz Bayesian criterion for clustering, and automatic identification of clusters (up to 15). The seven input variables into this cluster analysis were the proportions of usage in each of the seven acoustical environments. Three clusters were identified, and the silhouette measure of cohesion and separation for the three clusters was 0.4, considered to be *fair* cluster quality (≥ 0.5 considered *good*, which is highest cluster quality rating; SPSS Version 24).

**Figure 4.**
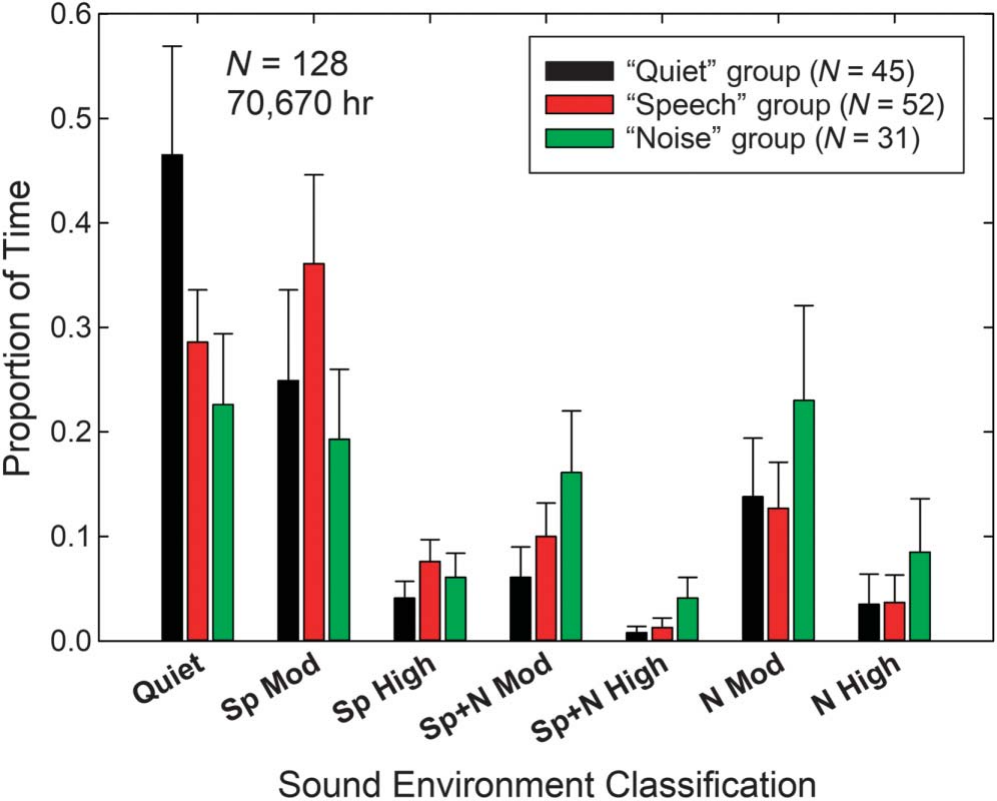
The unique environmental sound profiles of each of the three distinct subgroups of the 128 hearing aid wearers identified via cluster analysis. Based on these profiles, the subgroups were identified as “Quiet” (black), “Speech” (red), and “Noise” (green) groups. Sp = speech; N = noise, Mod = moderate level.

Figure 4 shows the means and standard deviations for the proportion of time spent in each acoustical environment for the participants in each of the three identified clusters or groups. We have labeled these three groups, based on the patterns in Figure 4, as the *Quiet* (black bars), *Speech* (red bars), and *Noise* (green bars) groups. The Quiet group, representing about 35% of the wearers, spent almost half of their time in environments classified as quiet, close to twice the proportion of time spent in such environments as either of the other two groups. The Speech group, representing 41% of the wearers, had the highest proportions of usage in environments involving speech only, both moderate and loud speech. Finally, the Noise group (24% of wearers) had the highest proportions of usage in each of the four environments involving noise. Seven univariate analyses of variance were performed, one for each of the seven sound environments, with the classified cluster membership serving as the sole independent variable and the proportion of time in that environment as the dependent variable. This was followed by three independent-samples pairwise *t* tests, with Bonferroni corrections, to examine group effects in more detail. All seven analyses of variance were significant, smallest *F*(2,125) = 24.831, *p* < .001, all post hoc pairwise *t* tests were significant (*p* < .05), except the Quiet versus Speech group differences for speech + noise high, noise moderate, and noise high environments. In these three environments, all of which included noise, the Noise group spent significantly greater proportions of time in such environments than the other two groups. This pattern of usage across environments led to the designation of these 31 wearers as the “Noise group.”

Do these three groups differ in some demographic or audiologic measure that might explain their differing patterns of environmental usage? To address this, we conducted a series of univariate analyses of variance on age, average high-frequency (1000, 2000, 4000 Hz) hearing loss in both ears, duration of hearing loss in years, years retired, score on a dementia screen, the Mini-Mental State Examination (Folstein, Folstein, White, & Messer, 2010), unaided score on the Hearing Handicap Inventory for the Elderly (Ventry & Weinstein, 1982), unaided sound-field speech recognition performance in noise, as well as aided measures of hearing aid performance, including aided Hearing Handicap Inventory for the Elderly, the Profile of Hearing Aid Performance (Cox & Gilmore, 1990), and aided speech recognition in noise. No significant differences were observed across the three environmental profile groups, largest *F*(2, 125) = 2.09, *p* = .13. In similar fashion, a series of Pearson chi-square analyses were conducted for a variety of categorical data from case histories for the three groups. The variables in these analyses were gender, highest level of education completed, total household income, living arrangements at home, frequency of social activities, and frequency of attending outside events. Here, one group comparison emerged as significant, χ^2^(4) = 11.5, *p* < .05: the frequency of attending social activities. Social activities on the case history form were defined as “visiting with a friend or in a group, entertaining in your home, eating in a restaurant, playing cards, etc.” There were three response alternatives for this item: “regularly participate…(almost daily),” “occasionally participate…(weekly),” and “rarely participate…(monthly or less).” About 49% of the Quiet group participated in such activities regularly, whereas a strong majority of the other two groups did so (75% of the Speech group and 81% of the Noise group). Importantly, the case histories were obtained prior to being fit for the first time with the hearing aids in this study. Thus, the acoustic environment profile that emerged for each group (see Figure 4) for aided listening appears to fit their social activity patterns as described prior to wearing hearing aids. Again, the three groups did not differ on any characteristics describing the individuals wearing the hearing aids, such as their age, hearing loss, gender, education level, or cognitive status. Rather, the three different patterns or clusters of environmental usage appear to be driven by the wearer's social activities more than some characteristic of the person. This is somewhat consistent with the findings of Wu and Bentler (2012) who found the wearer's social activities to influence the acoustic environments logged, although they found this to be driven largely by age differences among their participants. Perhaps, however, with an even greater range of measures, including personality, individual characteristics, such as introversion or extroversion, may be found to drive involvement in social activities and, as a result, the acoustic characteristics of the environments most frequently encountered by the wearers.

We also examined the influence of sound environment classification pattern on overall usage of hearing aids. The mean daily usage (total hours used bilaterally divided by the number of calendar days lapsed during datalogging) for the 128 wearers was 6.9 hr. The effect of environmental classification pattern on daily usage was significant, *F*(2, 124) = 4.89, *p* < .01, with post hoc pairwise comparisons revealing that the Noise group had significantly less (*M* = 5.1 h; *p* < .05) daily usage than both the Quiet (*M* = 7.7 h) and Speech (*M* = 7.2 h) groups. Those who wore their hearing aids in noisier conditions tended to wear their hearing aids less on a daily basis.

### Data From 65 Wearers Using Their Devices for an Average of 13 Months

**Figure 5.**
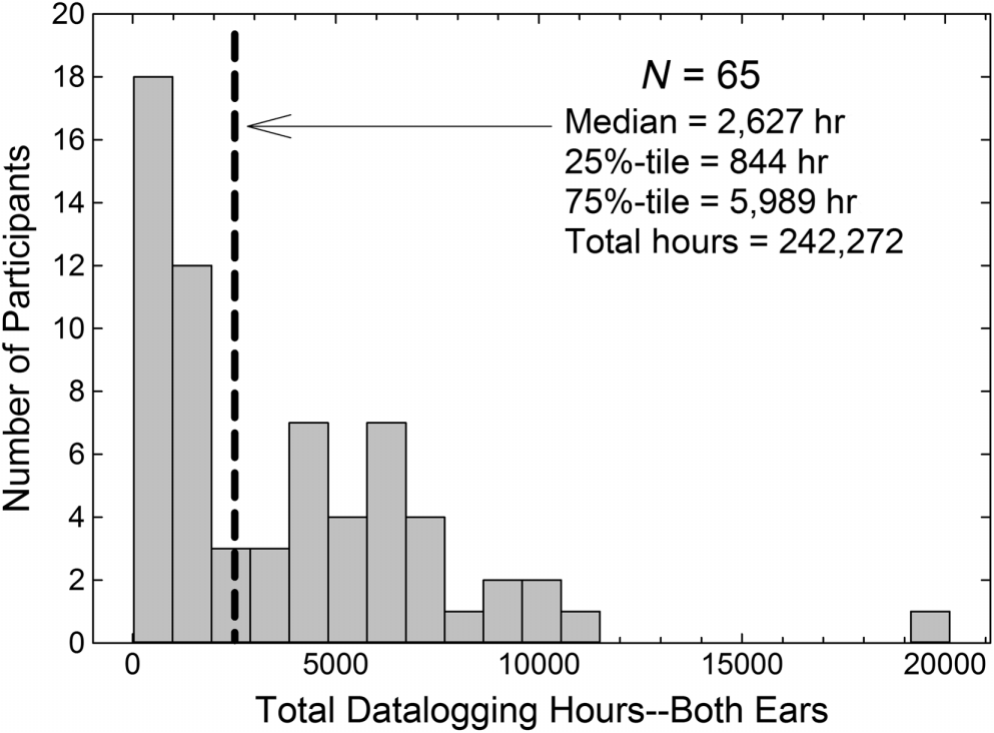
Histogram showing number of participants among the group of 65 who wore their hearing aids for a bilateral total of 0–999, 1,000–1,999, 2,000–2,999,…20,000–20,999 hr. The median value of 2,627 hr (~1,314 hr/ear) is shown as the heavy dashed line.

Figure 5 shows the distribution of total hours of usage logged for these 65 wearers who voluntarily returned for follow-up. The correlation between the total hours logged by the right and left devices was *r* = .98 (*p* < .001) supporting the reliability of these data and their pooling as total hours logged. The total hours logged for the entire group over time is 242,272 hr with that approximately evenly divided between the right (121,133 hr) and left (121,139 hr) devices. In terms of calendar months lapsed for the period of usage examined here, the median value was 13.3 months with a range of 4.5 to 39.4 months. For the participant with 20,112 total hours, about 10,000 per device, represented at the far right in Figure 5, the device was worn for 3.02 years for an average of 9.1 hr/day. This participant was one of three who had measurement durations of 2 years or more. As noted, most of the 65 were assessed following about 1 year of wear time (50% of wearers had 0.85–1.15 years wear time).

**Figure 6.**
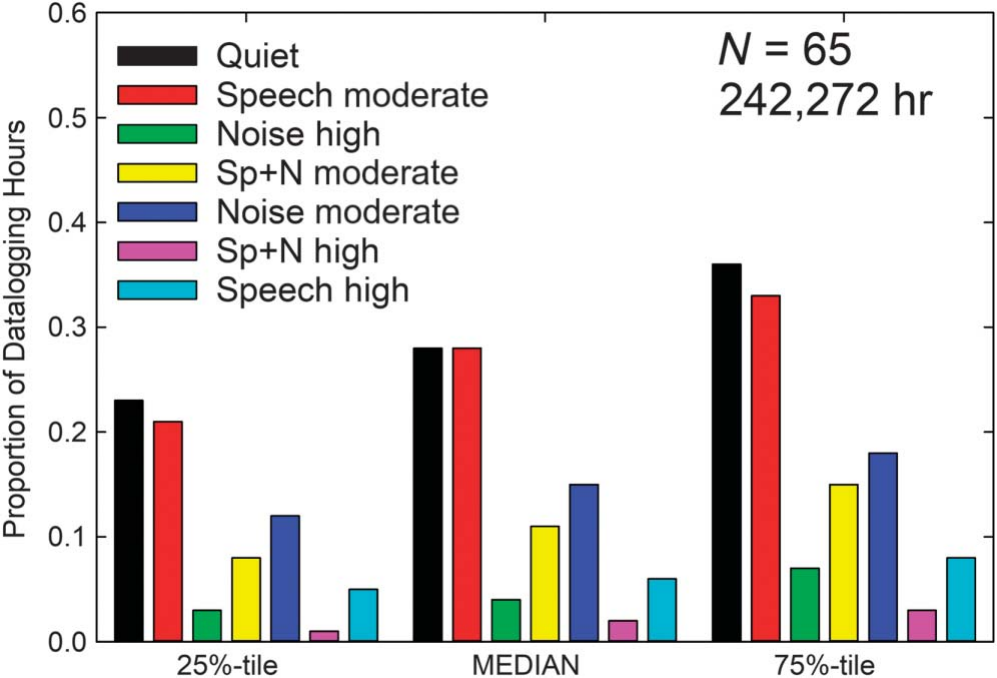
Medians and quartiles for the proportion of total hours the hearing aids were worn in each of seven acoustic environments as classified by the devices automatically for the 65 wearers who logged a total of 242,272 hr (summed across ears and participants). Sp+N = speech and noise.

Figure 6 shows the median and quartiles for the distribution of classified sound environments for these 65 wearers. Of these 65, 56 were among the 128 wearers whose data were analyzed above, with 43% being members of the Quiet group, 34% from the Speech group, and 23% from the Noise group. The distribution of these groups is like that of the larger group of 128 wearers, suggesting that the composition of this 65-member group is representative of that larger sample. As noted previously in the larger sample of wearers over a shorter duration (see Figure 3), the median wearer spent nearly 60% of the time in acoustic environments classified as quiet or moderate speech alone. Both are very favorable listening environments. The environment classifications were again found to be reliable, based on correlations between the right and left devices. Of the 65 wearers, 61 had archived datalogging data available for both the left and right devices. Of these 61, 54 had Pearson correlations between environment classification proportion profiles ≥ 0.90, and of the seven wearers with lower correlations, only two were < 0.70. Thus, over this much longer duration of usage, average wear time of 13 months versus 6 weeks, the reliability of the environmental classifications remains high.

**Figure 7.**
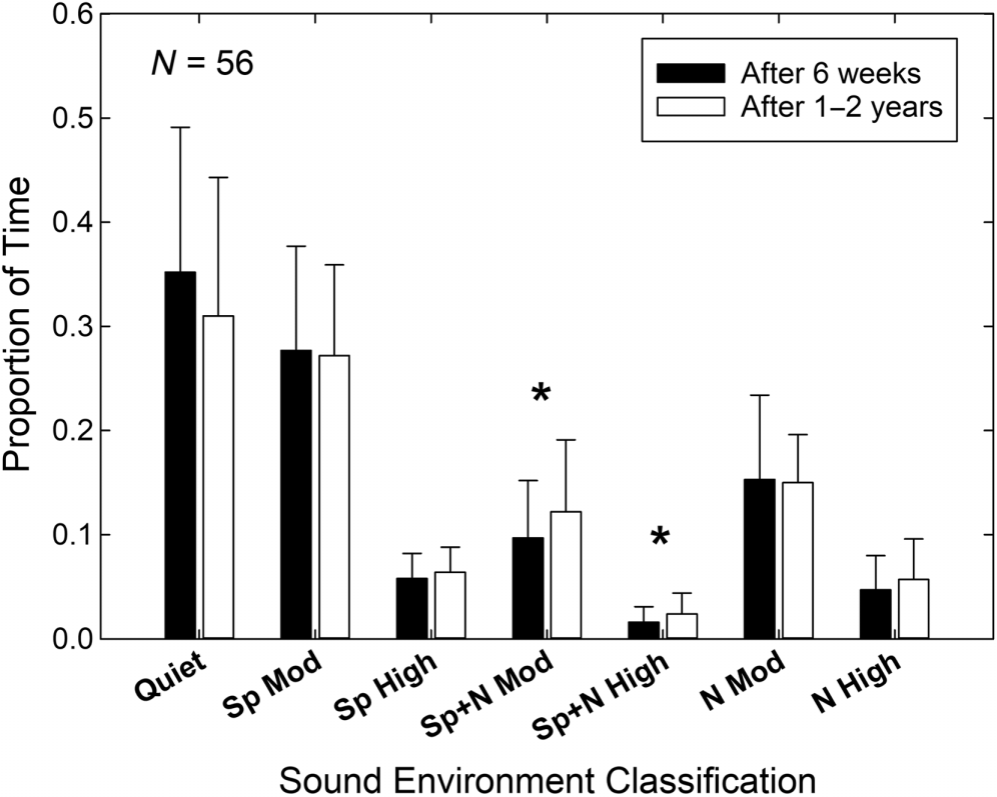
Comparison of sound environment profiles for 56 of the 65 wearers for whom datalogging information was available at both the “6-week” (black bars) and “13-month” (1–2 years; white bars) intervals. Significant differences are marked by asterisks. Sp = speech; N = noise; Mod = m sound level (< 75 dB SPL).

To examine the possible changes in listening environments over time by older adults with ARHL who wear hearing aids, we selected the 56 participants who had provided datalogging information for both the 6-week and 13-month periods. Figure 7 provides the sound environment profiles for these 56 wearers for the first 6 weeks of usage and over a usage period of 13 months (1–2 years). Paired *t* tests, *t*(55), were calculated. Using a Bonferroni-corrected significance level of *p* = .007 (or 0.05÷7), only the two environments with speech and noise changed significantly, although the proportion of time in quiet approached significance (*p* = .009). Over time, it appears that these 56 wearers increased the proportion of time spent in moderate and high levels of speech-and-noise, with this increase largely offset by less time spent in quiet conditions. Overall, however, the picture that emerges from the data in Figure 7 is one of a largely stable set of acoustical environments in which the devices are worn. This was also apparent for the individual data as evidenced by moderate-to-strong and statistically significant Pearson *r* correlations for the proportions of time spent in each environment over each period of usage. Only one of the seven correlations was weak (*r* = .26) and nonsignificant (*p* = .06). The other six were between 0.45 and 0.71 and statistically significant (*p* < .001). Thus, on both a group and individual basis, the acoustical environments in which older adults with ARHL wear their hearing aids appear to be quite stable through the first year of usage.

We also examined the average daily usage in hours per day over time. After 6 weeks of usage, these 65 older adults with ARHL wore their hearing aids for an average (*M*) of 7.0 hr per day (*SD* = 3.7 hr per day). A paired *t* test showed that the decline to a mean of 4.4 hr per day (*SD* = 3.7 hr per day) over the first year or so of usage was statistically significant, *t*(64) = −5.8, *p* < .001. The Pearson correlation between the average usage over the first 6 weeks and the subsequent year was *r* = .54 (*p* < .001), indicating that the general trend for the 65 participants was for daily usage to decrease over time. Significant declines over a year of hearing aid usage have been observed previously in older adults with ARHL (Humes, Wilson, Barlow, Garner, & Amos, 2002).

Regardless, 4–7 hr per day represents only a portion of the typical older adult's day during which hearing aids could be worn. Thus, despite capturing sound environment data for tens of thousands of hours of hearing aid usage by older adults with ARHL, this may still only represent a small portion of the acoustic environments in which the wearer is immersed. Moreover, the older adults in this study could have simply elected to wear their hearing aids only in those environments in which they thought the hearing aids would work well. Thus, these data provide insights into the acoustic environments in which older adults with ARHL choose to wear their hearing aids but not necessarily all such environments encountered daily.

These sound classification data are largely consistent with those from the studies reviewed in the introduction. It appears that older adults with hearing aids generally choose to wear their devices in favorable listening conditions, such as quiet and speech only. When noise is present, it tends to be moderate in level, which likely means a favorable signal-to-noise ratio (Pearsons et al., 1977; Smeds, Wolters, & Rung, 2015). This is true despite all the previous data coming from older adults with impaired hearing who were experienced hearing aid wearers and all participants in this report being new hearing aid users at the outset. Nonetheless, within the same group of wearers, there were some systematic changes in the sound environments encountered by hearing aid wearers over the first year.

The data presented in this report are clearly dependent on the validity of the particular environment classification system used in the study hearing aids. The validity of this sound classifier has had only limited investigation. As noted, the manufacturer of the study hearing aids has published a validation study (Groth & Cui, 2017), although not in a peer-reviewed journal. There was some additional validation in this study in that those who were identified through their sound classification profiles as preferring quiet listening conditions also tended to indicate a preference for such conditions in a prestudy survey. Further, the fact that the findings from these two more extensive datasets agree with those reviewed in the introduction that were obtained with other methods can also be considered a form of validation. Nonetheless, further validation of the sound classification system used here, as well as those in devices from other manufacturers, is warranted with publication of the validation in the peer-reviewed literature.

## Summary

Datalogging results for the automatic classification of sound environments regarding the presence of speech, noise or both, and sound of either moderate (< 75 dB SPL) or high (≥ 75 dB SPL) levels, were presented from tens of thousands of hours of usage by older adults with ARHL wearing hearing aids for the first time. The general picture that emerged was that hearing aids were worn in favorable listening conditions most of the time by these older adults. The measurements appeared to be reliable in that two independent devices, one on each ear, provided highly correlated datalogging results. Moreover, three distinct patterns of exposure to acoustic environments emerged, with participants following each pattern labeled here as the *Quiet*, *Speech,* and *Noise* groups. The automatic classification of the sound environment was also related to the wearer's social activities, with those having fewer such activities yielding environment classification results indicating a higher proportion of the time spent in quiet acoustic environments. This can be considered a form of validation for the automatic classifier used here as well. Finally, the pattern of acoustic environments experienced by the wearers, most found to be quite favorable, was also found to be stable over an average period of usage of 13 months, although the daily usage in hours/day declined considerably over this period for these first-time wearers.
